# P_5A_-Type ATPase Cta4p Is Essential for Ca^2+^ Transport in the Endoplasmic Reticulum of *Schizosaccharomyces pombe*

**DOI:** 10.1371/journal.pone.0027843

**Published:** 2011-11-21

**Authors:** Ana Cristina D. M. Lustoza, Livia M. Palma, Arnoldo R. Façanha, Lev A. Okorokov, Anna L. Okorokova-Façanha

**Affiliations:** 1 Laboratório de Fisiologia e Bioquímica de Microrganismos, Centro de Biociências e Biotecnologia, Universidade Estadual do Norte Fluminense Darcy Ribeiro, Rio de Janeiro, Brazil; 2 Laboratório de Biologia Celular e Tecidual, Centro de Biociências e Biotecnologia, Universidade Estadual do Norte Fluminense Darcy Ribeiro, Rio de Janeiro, Brazil; Newcastle University, United Kingdom

## Abstract

This study establishes the role of P_5A_-type Cta4 ATPase in Ca^2+^ sequestration in the endoplasmic reticulum by detecting an ATP-dependent, vanadate-sensitive and FCCP insensitive ^45^Ca^2+^-transport in fission yeast membranes isolated by cellular fractionation. Specifically, the Ca^2+^-ATPase transport activity was decreased in ER membranes isolated from cells lacking a *cta4^+^* gene. Furthermore, a disruption of *cta4^+^* resulted in 6-fold increase of intracellular Ca^2+^ levels, sensitivity towards accumulation of misfolded proteins in ER and ER stress, stimulation of the calcineurin phosphatase activity and vacuolar Ca^2+^ pumping. These data provide compelling biochemical evidence for a P_5A_-type Cta4 ATPase as an essential component of Ca^2+^ transport system and signaling network which regulate, in conjunction with calcineurin, the ER functionality in fission yeast.

## Introduction

Calcium plays a key role in signal transduction in eukaryotic cells and modulates a variety of cellular functions. The calcium signaling is initiated by opening the Ca^2+^ channels located at plasma membrane and membranes of the organelles [1]–[3] which leads to transient and local increase in the concentration of cytosolic calcium ions ([Ca^2+^]i) ten to hundreds times above the basal level, followed by closing the channels. The calcium signal is terminated when cytosolic-free Ca^2+^ concentration is reduced to basal levels by Ca^2+^-ATPases and Ca^2+^/H^+^ exchangers that transport calcium out of the cell or sequester it in the organelles [4]. Endoplasmic reticulum (ER) plays a crucial role in calcium sequestering and signaling. High concentrations of Ca^2+^ ions are required for the activities of numerous enzymes that catalyze the folding, modification, processing and trafficking of secretory proteins [4], [5]. Different stimuli can cause disruption of ER function including calcium depletion from the ER lumen, inhibition of protein glycosylation, reduction of disulfide bonds, which all can affect the efficiency of protein folding and cause accumulation of unfolded proteins in the ER. A first response to this ER stress is the stimulation of a signaling pathway appropriately termed the unfolded protein response that selectively activates transcription of the genes encoding ER-resident molecular chaperones [6]–[8]. Evidences indicate that one of the responses to ER stress is a stimulation of Ca^2+^ influx at the plasma membrane that serves to replenish the Ca^2+^-depleted organelle and to trigger calcium signaling pathways in both animal and yeast cells [9], [10]. Ca^2+^/calmodulin-dependent protein phosphatase or calcineurin which is required for cytokinesis and ion homeostasis [11], [12] was shown to be activated during ER stress [13]–[15].

In animal cells, the calcium transport to endoplasmic/sarcoplasmic reticulum is mediated by SERCA Ca^2+^-ATPases which belong to the P_2A_ subfamily of P-type ATPases [16], [17]. Yeast cells lack homologs of SERCA pumps. We and others showed by means of fluorescence microscopy that the members of P_5A_-type ATPases were localized to yeast ER, namely Cta4p ATPase of the fission yeast *Schizosaccharomyces pombe* [18], and Spf1/Cod1 ATPase from *Saccharomyces cerevisiae* [19]–[20], which share 49% amino acid sequence identity. In spite of indirect indications that P_5A_-ATPases are involved in calcium homeostasis [18], [20] the substrate specificity of these pumps remains unassigned. Purified Spf1p/Cod1p was used for determination of ATPase activity *in vitro* in the presence of several cations including Ca^2+^, however, they failed to stimulate hydrolytic activity [20]. The authors suggested that the factors coupling Spf1p/Cod1p to a specific ion might be lost during the purification of the enzyme from *S. cerevisiae* membranes [20]. It appears that the *S. cerevisiae* Spf1p/Cod1p ATPase forms an oligomeric endomembrane complex [21]. In this case, the biochemical evidence for the P_5A_ ATPase as a Ca^2+^-ATPase should be obtained from the measurements of ATP-dependent Ca^2+^ transport activity in native yeast membranes and comparison between wild type and null mutant cells. However, till now, there were no reports on Ca^2+^-ATPase activity in fission yeast membranes.

Previous data show that the absence of ER located yeast P_5A_-ATPases leads to sensitivity towards calcium stress [18], [20] and changes in nuclear calcium levels [18]. These data provided impetus for the current study, which addressed the role of P_5A_-type Cta4 ATPase in controlling ER Ca^2+^ store and signaling in *S. pombe*.

## Methods

### Yeast strains and growth conditions

Strains of *Schizosaccharomyces pombe* used in this study were the wild type Fy1180 (*h^+^ otr1*R(*Sph*I)::*ade6^+^ ura4-D18 leu1-32 ade6-*M210), the strain Hu185 expressing GFP-tagged Cta4p (*h^+^ cta4-GFP::kanMX6 otr1R(SphI)::ade6* ^+^ *ura4-D18 leu1-32 ade6-*M210), and the mutant strain Hu285 lacking Cta4p (*h^−^ cta4::ura4^+^ ura4-D18 leu1-32 ade6-*M216) [18]. The fission yeast strains were grown in standard YES medium at 30°C containing 0.5% yeast extract and 3% glucose supplemented with adenine, uracil, arginine, histidine and leucine (75 mg/L). For spot assays, yeast cells were serially diluted in five-fold steps, spotted onto YES plates containing DTT (0.4%) or tunicamycin (0.05 µg/mL).

### ^45^Ca^2+^ accumulation measurements in yeast cells

Total cellular accumulation of Ca^2+^ in yeast cells (cytosol and organelles) was measured as described [22]. Briefly, yeast cells were grown to log phase in YES medium, harvested and resuspended in fresh YES medium supplemented with tracer quantities (110.8 µg/mL; 2.5 mCi/mL, Amersham Pharmacia) of ^45^CaCl_2_. After 5 h incubation at 30°C, cells were harvested rapidly by filtration onto GF/F filters (Whatman), washed with ice-cold buffer (10 mM CaCl_2_, 5 mM MES-NaOH pH 6.5), placed in scintillation vials and processed for liquid scintillation counting on a liquid scintillation counter. The specific activity of the culture medium was determined in each experiment. Cell optical density was determined at 600 nm.

### Membrane isolation and fractionation

Yeast membranes were isolated and fractionated according to [23], [24]. Briefly, the middle logarithmic phase cells were transformed to spheroplasts by incubation at 37°C in buffer containing 1.2 M sorbitol, 10 mM Tris-HCl, pH 7.4, 30 mM β-mercaptoethanol and 5 mg of lytic enzymes from *Trichoderma* (Sigma)/1 g of wet cells. After 50 min the incubation mixture of spheroplasts, old cells and cell walls was rapidly cooled and received EDTA, benzamidine and PMSF at 1.2 M sorbitol and 10 mM Tris–HCl, pH 7.4 to give final concentrations 1 mM of each protease inhibitor. The obtained mixture was added to a solution of 1.4 M sorbitol in 10 mM Tris-HCl, pH 7.4, centrifuged for 5 min at 3,000×*g* and then resuspended and homogenized in a Potter glass homogenizer using a lysis buffer (12.5% sucrose, 20 mM MOPS-Na pH7.4, 1 mM DTT, 1 mM benzamidine, 1 mM PMSF and a cocktail of the polypeptide protease inhibitors). Total membranes were precipitated for 45 min at 120,000×*g*, resuspended in lysis buffer and loaded onto a 12-step gradient formed of 56, 52, 48, 45, 42, 39, 36, 33, 30, 25 and 20% sucrose (w/w) prepared in 10 mM MOPS-Na pH 7.2. The cocktail of the polypeptide protease inhibitors was applied to each step of gradient. After centrifugation for 2 h 45 min at 140,000×*g* membrane fractions were collected from the bottom and frozen. A 3-step sucrose gradient was formed of 50, 38 and 25% sucrose (w/w); membrane fractions enriched with vacuole, Golgi, ER/nuclear envelope were collected from the respective interfaces after centrifugation for 2 h 45 min at 140,000×*g* [23].

Activities of organellar marker enzymes (NADPH cytochrome c oxidoreductase and GDPase) as well as the protein determination followed already published procedures [23]–[26]). Sucrose concentration was determined using a refractometer. To identify membrane fractions enriched with nuclear membranes, the fission yeast strain with constitutive accumulation of the transcription factor Pap1/Caf3 in the nucleus was used [27] and distribution of the GFP-tagged Pap1/Caf3-89 was analyzed by immunoblot.

### ^45^Ca^2+^ transport in membrane vesicles

^45^Ca^2+^ uptake by isolated membranes vesicles was measured by the filtration method [25]. The standard incubation mixture contained 10 mM Tris-HCl pH 7.2; 160 mM KCl, 5 mM MgCl_2_, 1 mM ATP, 9.8 µM EGTA, 10.4 µM CaCl_2_, 0.5 µCi ^45^Ca^2+^ (Amersham Life Sciences) and 2 µM cyanide *p*-(trifluoromethoxy)phenyl-hydrazone (FCCP). At concentration of Cl^−^ used in assay (160 mMKCl)) the membrane potential is converted into ΔpH from E_M_^H+^ or to ΔpCa^2+^ from E_M_^Ca2+^. The reaction was initiated by adding 10–30 µL of suspension of total membrane vesicles or membrane fractions. After incubation at 30°C, 180 µL aliquots were injected into 10 mL of stop solution (150 mM KCl, 5 mM MgCl_2_, 10 mM MOPS-KOH, pH 7.2), filtered on 0.45 µm nitrocellulose filters (Millipore) and washed with 10 mL of the same buffer. Radioactivity retained on the filters was measured by scintillation counting. The specific activity of the reaction medium was determined in each experiment. The values were normalized per mg of total membrane protein.

### Immunoanalysis

Yeast membranes from the sucrose gradient fractions (10 µL) and total membranes (5 µL, 20 µg/µL and 6 µg/µL of wt and mutant, respectively) were spotted on nitrocellulose membranes and probed with anti-BiP (dilution 1∶5,000) or anti-GFP antibodies (1∶400). Anti-GFP antibodies were purchased from Sigma-Aldrich. Anti-BiP antibodies were provided by Prof. J. Armstrong (University of Sussex, Brighton, UK). The blots were developed with peroxidase-conjugated secondary antibody (GE Healthcare).

### Protein extraction and calcineurin phosphatase activity assay

Spheroplasts were obtained and lysed as described above for membrane isolation. The homogenate was passed through a Sephadex G-25 column to remove the free phosphate. The solution eluted from the column was used in the calcineurin phosphatase activity assay. Calcineurin protein phosphatase activity was assayed using the Calcineurin Cellular Activity Assay Kit (Calbiochem) according to the manufacturer's instruction. The assay was carried out in 50 µL volumes in a microtiter plate using RII phosphopeptide, the most efficient and commonly used peptide substrate for calcineurin. The reaction was started by adding the cellular extracts, followed by incubation at 30°C for 30 min, and terminated by adding the Malachite Green reagent according to the manufacturer's instruction. The activity of calcineurin protein phosphatase was determined by the amount of free phosphate released and normalized per mg of protein.

## Results

### Loss of Cta4 ATPase results in increased intracelullar Ca^2+^ accumulation

**Figure 1:**
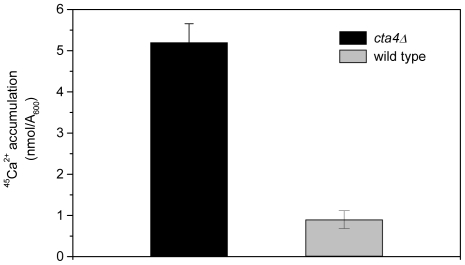
Loss of *cta4^+^* results in elevated cellular Ca^2+^ levels. ^45^Ca^2+^ accumulation in wild-type cells and mutant cells lacking Cta4 ATPase was measured after incubation for 5 hr in standard medium supplemented with ^45^Ca^2+^ as described in Materials and Methods. Values are means (± SE) of three independent experiments.

To address the role of Cta4 ATPase in Ca^2+^ homeostasis, we investigated the effect of *cta4^+^* deletion on cellular calcium levels. We found that mutant cells lacking Cta4 ATPase exhibited 6-fold increase in total calcium accumulation as compared to wild-type cells (Figure 1). This result suggests that loss of Cta4 ATPase leads to enhanced calcium influx which might occur in response to depletion of Ca^2+^ from secretory organelles.

### ATP-dependent Ca^2+^ transport is reduced in *cta4Δ* membranes

**Figure 2:**
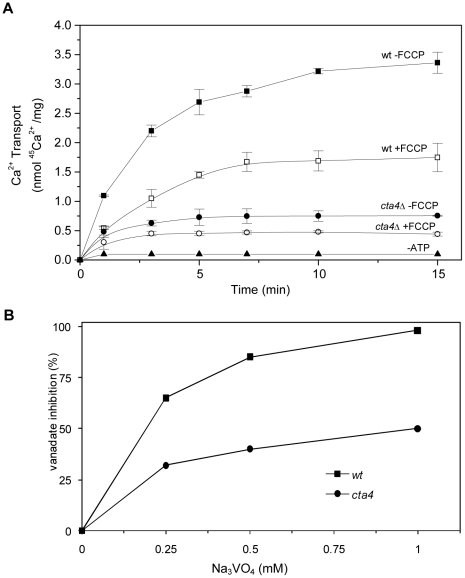
^45^Ca^2+^ uptake by total membranes of *S. pombe* is mediated by Ca^2+^- ATPases and Ca^2+^/H^+^ exchangers. Total membranes were isolated from wild type (▪,□) and *cta4Δ* (•,○) cells and subjected to determination of ^45^Ca^2+^ transport in the presence of 1 mM ATP and in the presence (□,○) or absence (▪,•) of 2 µM FCCP (A). Inhibition of ATP-dependent FCCP-insensitive ^45^Ca^2+^ transport by vanadate (Na_3_VO_4_), the inhibitor of P-type ATPases (B). Values are means (± SE) of four independent experiments.

To examine whether Cta4p participates in Ca^2+^ sequestering along the secretory pathway, we measured at first the ATP-dependent ^45^Ca^2+^ transport in total membrane vesicles isolated from wild-type and *cta4Δ* mutant cells. In order to distinguish between Ca^2+^ transport mediated by Ca^2+^-ATPases and Ca^2+^/H^+^ exchangers, protonophore FCCP was used to collapse the transmembrane H^+^ gradient and eliminate the Ca^2+^/H^+^ exchanger component of transport. As shown in Figure 2A, Ca^2+^ transport in total membranes isolated from wild-type cells was decreased by 50% upon FCCP addition (see 15 min point), indicating that remaining 50% of the transport was mediated by Ca^2+^-ATPases. Indeed, this ATP-dependent FCCP-insensitive ^45^Ca^2+^ transport was also sensitive to orthovanadate, the specific inhibitor of P-type ATPases, showing 85% and 98% inhibition at 0.5 mM and 1 mM, respectively (Figure 2B).

The loss of Cta4p resulted in strong reduction (4.5-fold) of total ATP-dependent ^45^Ca^2+^ transport (Figure 2A) of which only 40% was FCCP-sensitive, reflecting lower activity of Ca^2+^/H^+^ antiporters in mutant cells. ^45^Ca^2+^ transport mediated by Ca^2+^-ATPase (FCCP-insensitive transport) was 4-fold lower in *cta4Δ* membranes than in wild type (Figure 2A) and 50% of this activity was vanadate-sensitive with IC_50_∼0.2 mM (Figure 2B). These data clearly indicate that *cta4^+^* is required for Ca^2+^-ATPase activity and Ca^2+^ sequestering in fission yeast membranes.

### Cta4 ATPase is required for Ca^2+^ transport in ER membranes vesicles

To identify the membrane fractions responsible for Cta4p-dependent Ca^2+^ uptake, separation of the fission yeast membranes on a 12-step sucrose gradient was performed. Determination of sucrose density in addition to biochemical and immunochemical characterization showed that membrane fractions 1–11 were likely derived from the nucleus according to GFP-tagged Caf3-89 immunodetection (not shown, [27]). Fractions 12–28 and 29–38 were enriched with the ER and Golgi, respectively, as revealed by analysis of BiP and Cta4p∼GFP distribution, and GDPase activity; vacuoles migrated with light membrane vesicles in fractions 39–50 [23].

**Figure 3:**
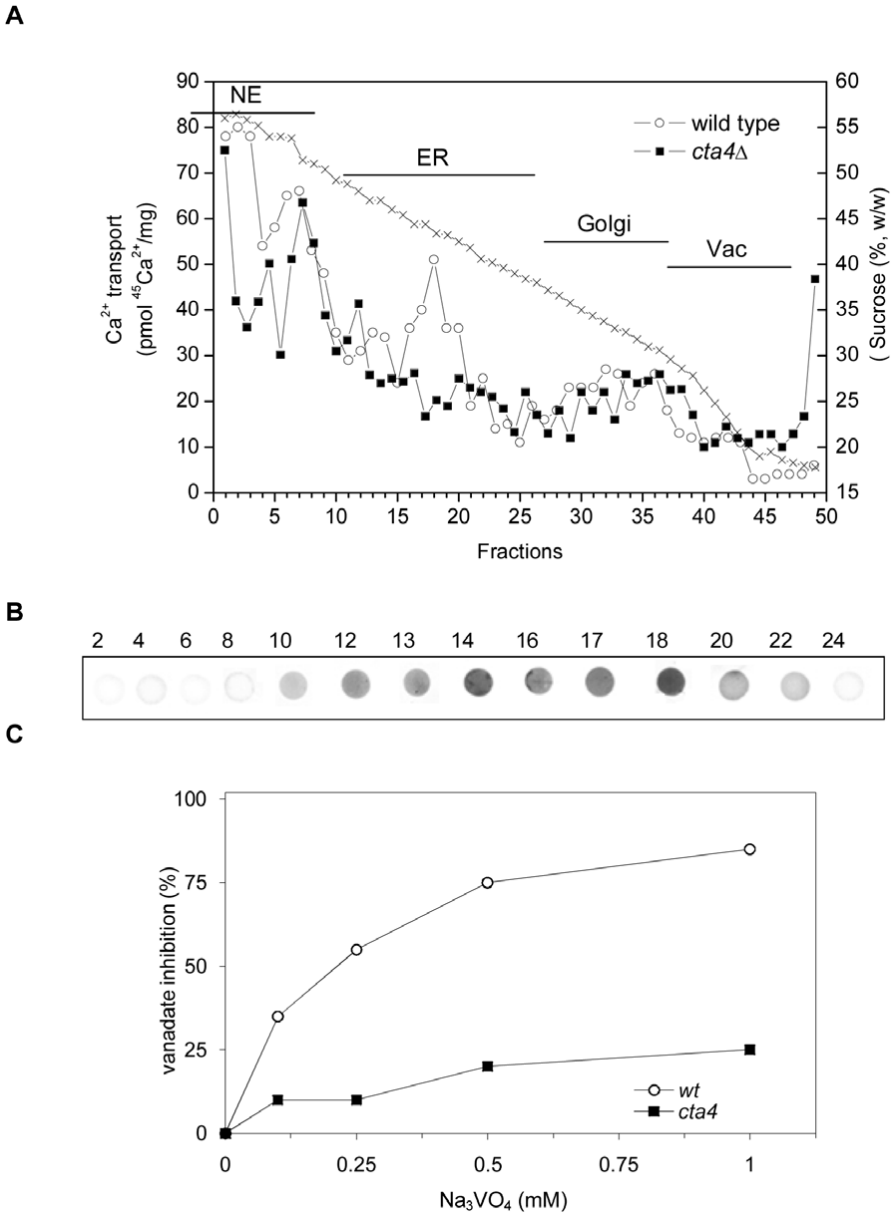
*cta4^+^* is required for Ca^2+^-ATPase activity in ER membrane fractions. Total membranes were isolated from wt and *cta4Δ* cells and fractionated on 12-step sucrose density gradient. (A) ATP-dependent FCCP-insensitive ^45^Ca^2+^ uptake in the membrane fractions was measured after 10 min of incubation as described in Materials and Methods. Sucrose concentration of each fraction is shown. (B) Dotblot of selected gradient fractions (fraction numbers are indicated) was used for immunolocalization of Cta4-GFP using anti-GFP antibodies. (C) Inhibition of ATP-dependent FCCP-insensitive ^45^Ca^2+^ transport in ER membrane fractions by vanadate (Na_3_VO_4_), the inhibitor of P-type ATPases. Results shown are representative of three independent experiments. Abbreviation used: NE, nuclear envelope; ER, endoplasmic reticulum; Vac, vacuole.

ATP-dependent FCCP-insensitive ^45^Ca^2+^ transport was detected along the sucrose gradient in different membrane fractions indicating the presence of Ca^2+^ pumps in various compartments of *S. pombe* (Figure 3A). The highest Ca^2+^-ATPase activities were detected in ER, nucleus and Golgi membranes. Comparative analysis revealed that the peak of Ca^2+^-ATPase activity was absent in ER membrane fractions 15–21 of *cta4Δ* (Figure 3A, closed symbols). In agreement, the corresponding fractions 15–22 isolated from the fission yeast strain expressing GFP-tagged Cta4p were immuno-reactive with anti-GFP antibodies (Figure 3B and [18]), indicating the dependence of Ca^2+^ transport in these membrane vesicles on *cta4^+^*. In addition, Ca^2+^-ATPase transport activity in ER fractions of wild type was significantly inhibited by vanadate (85%, 1 mM) whereas most of this vanadate-sensitive activity disappeared in ER fraction of *cta4Δ* (Figure 3C).

It is noteworthy that although ATP-dependent FCCP-insensitive ^45^Ca^2+^ transport was reduced in ER and membranes heavier than ER (mainly nucleus) of *cta4Δ*, it was augmented in vacuolar fractions (Figure 3A).

**Figure 4:**
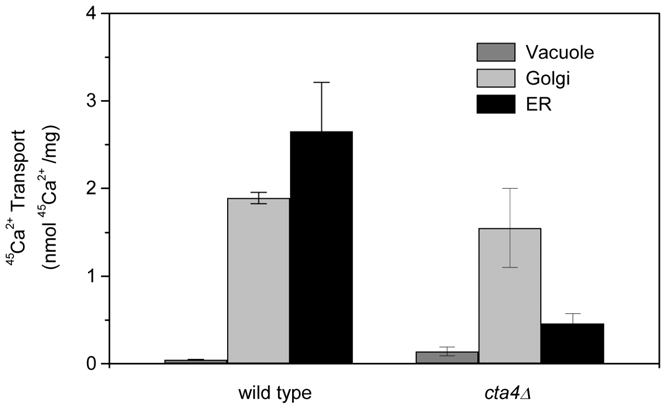
Loss of Cta4 ATPase results in reduced Ca^2+^ pumping in endoplasmic reticulum. Total membranes were isolated from wt and *cta4Δ* mutant cells and then fractionated on 3-step sucrose density gradient as described in Materials and Methods. Fractions were subjected to determination of the 10 min ^45^Ca^2+^ uptake in the presence of 2 µM FCCP and 1 mM ATP. Values are means (± SE) of four independent experiments.

These results were further confirmed using 3-step fractionation yielding three membrane fractions enriched with vacuole, Golgi and ER/nuclear vesicles. ER/N membranes of wild-type cells exhibited 6-fold higher Ca^2+^-ATPase transport activity as compared to the membranes lacking Cta4p (Figure 4). The deletion of *cta4^+^* resulted in up-regulation of Ca^2+^ pump activity in light membrane fraction reinforcing a compensatory role of vacuole enzyme.

Altogether, these results demonstrate that Cta4p expression is crucial for ATP-dependent Ca^2+^ transport in endoplasmic reticulum and that *cta4^+^* deletion has a profound effect on Ca^2+^ homeostasis within membrane network of fission yeast.

### ER stress response in *cta4* ^+^ deletion mutant and activation of calcium influx

**Figure 5:**
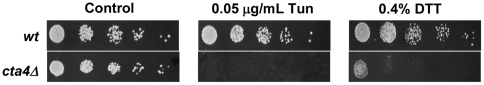
The growth of *cta4Δ* is impaired by endoplasmic reticulum stress. Wild-type and *cta4Δ* cells were serially diluted in five-fold steps, spotted onto YES plates containing 0.4% DTT and 0.05 µg/mL tunicamycin and incubated for 3 days at 30°C.

To explore how the *cta4^+^* disruption interferes with the ER function, we tested the sensitivity of yeast cells towards ER stress inducers DTT and tunicamycin which inhibit the protein disulfide bond formation and N-glycosylation, respectively. The mutant *cta4Δ* cells displayed sensitivity towards these substances while the growth of wild-type cells was not affected (Figure 5). Thus, the absence of Cta4 ATPase results in inability to cope with the accumulation of unfolded proteins.

**Figure 6:**
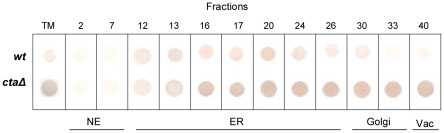
Cells lacking Cta4p exhibited higher levels of the ER stress indicator, BiP. Total membranes (TM) were isolated from yeast cells, fractionated on a sucrose density gradient and submitted to immunoblots analysis as described in Materials and Methods. Dot blots of individual fractions (10 µL, numbers are indicated) were used. Abbreviation used: NE, nuclear envelope; ER, endoplasmic reticulum; Vac, vacuole.

This assumption was further verified by detection of the ER chaperone BiP. In wild-type cells BiP was localized to membrane fractions which correspond to ER (Figure 6, fractions 12–26) and where GFP-tagged Cta4p was also detected (Figure 3A, fractions 8–22). On the other hand, the distribution pattern of BiP in *cta4Δ* differs from that of wild type occurring not only in ER but also in Golgi and vacuole membranes fractions (Figure 6, fractions 12–38). Also, BiP expression was significantly higher in *cta4Δ*. Hence, the lack of Cta4 ATPase triggers ER stress response and interferes with secretory pathway.

**Figure 7:**
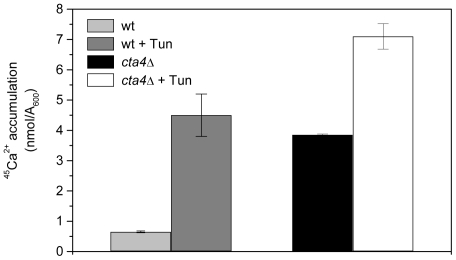
Endoplasmic reticulum stress caused by inhibition of glycosylation stimulates an increase of Ca^2+^ accumulation. The accumulation of ^45^Ca^2+^ in wild-type and *cta4Δ* yeast cells was measured after 5 hours of addition of ^45^Ca^2+^ to YES medium containing 0.125 µg/mL tunicamycin. Values are means (± SE) of three independent experiments.

Next, the calcium accumulation in *S. pombe* cells under ER stress was investigated. As shown in Figure 7, exposure of wild type cells to tunicamycin promoted 5-fold increase in ^45^Ca^2+^ accumulation reaching the levels of that of untreated *cta4*Δ cells, while *cta4Δ* exhibited only 2-fold stimulation. These findings reinforce a notion that ER function is severely impaired in the absence of Cta4 ATPase and suggest that enhanced calcium accumulation in *cta4Δ* might be explained as a result of the induction of ER stress response which, in turn, is related to disturbance of calcium transport in ER.

### Stimulated calcineurin controls calcium influx in cells lacking *cta4* ^+^

**Figure 8:**
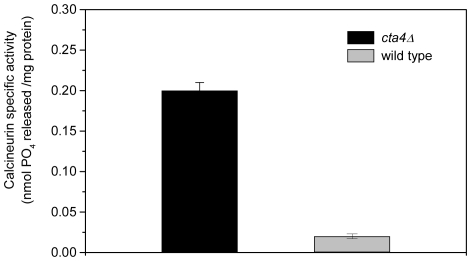
Mutant *cta4Δ* displays higher calcineurin phosphatase activity. The calcineurin protein phosphatase activity was determined by the amount of free phosphate released. The reaction was started by adding the cellular extracts, followed by incubation at 30°C for 30 min with the RII phosphopeptide substrate. Values are means (± SE) of three independent experiments.

**Figure 9:**
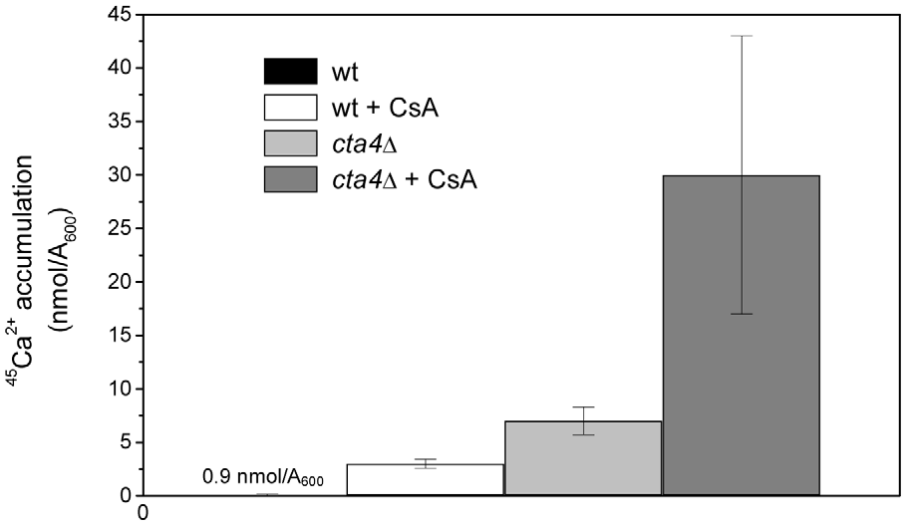
Calcineurin inhibition stimulated ^45^Ca^2+^ accumulation in wt and *cta4Δ* mutant cells. The accumulation of ^45^Ca^2+^ in wild-type and *cta4Δ* yeast cells was measured after 5 hours of addition of ^45^Ca^2+^ to YES medium containing 10 µg/mL cyclosporin A (CsA). Values are means (± SE) of three independent experiments.

It has been shown previously that *cta4Δ* is unable to grow in the presence of cyclosporine (CsA) [18], indicating that survival of the mutant cells requires Ca^2+^/CaM-dependent phosphatase calcineurin. Analysis of calcineurin specific activity in wild type and *cta4Δ* cells revealed that *cta4^+^* deletion resulted in drastic, 10-fold stimulation in calcineurin activity compared to wild-type cells (Figure 8). Furthermore, CsA treatment induced ^45^Ca^2+^ accumulation by 7-fold in *cta4Δ* mutant cells, reaching nearly 9-fold higher levels than that of wild type cells grown in the presence of calcineurin inhibitor and 30-fold higher than of wild type strain grown in standard conditions (Figure 9). These findings indicate that calcineurin negatively regulates calcium influx into fission yeast cells and performs essential function in *cta4Δ* preventing lethal elevation of intracellular calcium in response to ER dysfunction.

## Discussion

The members of P_5_-type ATPases are found in all eukaryotic genomes analyzed to date and present a number of striking features in their amino acid sequences and membrane topology that set them apart from the other enzymes of this type [17], [28], [29]. Previous studies based on indirect methods suggested the involvement of ER localized P_5A_ ATPases Cta4 and Spf1/Cod1 from fission and budding yeast, respectively, in calcium homeostasis [18], [20]. Heterologous expression of Arabidopsis MIA protein was shown to restore the growth of *spf1/cod1* null mutant on solid medium containing lovastatin [30]. Recent study in *S. pombe* implies the participation of a second P_5A_-ATPase in calcium and manganese homeostasis [31]. Unraveling the substrate specificities of P_5_ ATPases through biochemical means has increasingly been recognized as a major goal [32]. The analysis of Ca^2+^ transport in isolated fission yeast membranes represents a starting point for biochemical characterization of P_5A_-ATPase. Apart from Cta4p ATPase, two putative P_2_-type Ca^2+^-ATPases were revealed in fission yeast *S. pombe*, namely Pmc1p (SPAPB2B4.04c), and Pmr1p/Cps5p (SPBC31E1.02c) [28], [33]. Pmr1p localizes predominantly in the endoplasmic reticulum (ER) membrane while Pmc1p is limited to vacuole [33].

Using biochemical and genetic approaches, this study establishes the role of P_5A_-type Cta4 ATPase in Ca^2+^ sequestration in the ER. Our conclusion follows from the finding that ATP-dependent, FCCP-insensitive and vanadate-sensitive Ca^2+^ transport activity was severely diminished in ER membranes isolated from cells lacking Cta4 ATPase. To our knowledge, this is also the first report describing a Ca^2+^-ATPase transport activity in fission yeast endoplasmic reticulum, Golgi and vacuole membranes. For evolutionary reasons that remain unclear, *S. pombe* and *S. cerevisiae* lack homologs of SERCA pumps. In this scenario, another ATPase should assure Ca^2+^ transport in ER. The present study provides biochemical evidence that the P_5A_ Cta4 ATPase is essential for Ca^2+^-ATPase activity in ER and is crucial for establishing yeast ER homeostasis by regulating calcium transport. In our previous work only Ca^2+^/H^+^ exchange activity was measurable in membranes of *S. pombe* suggesting that Ca^2+^-ATPases were not expressed or not detected by unknown reasons [24]. Since both studies differ only by growth medium (peptone lacking YES in present study and YPD in former one), we assume that this factor can determine the effective expression of one or both types of the Ca^2+^-transporters.

Previous lack of evidence for the substrate specificity of P_5A_-ATPases led some authors propose that P_5A_-ATPases could be a phospholipid-ATPase mainly due to its homology with some flippases [34]. However, the available information on the biochemical properties of flippases from *S. cerevisiae* and other organisms does not support this alternative, since this enzymes shows a very low affinity for ATP (Km∼1.5 mM ATP [35]), in contrast to Km∼15 µM ATP found for Spf1/Cod1p [20] that is in the range of the high affinity for ATP described for most Ca^2+^-ATPases. In addition, flippases exhibit a very high sensitivity to vanadate with IC_50_ = 1–5 µM [35], [36] while Ca^2+^ transport in Cta4p-containing membranes was inhibited by 50% at 200 µM (Figures 2B and 3C), in the same way as Spf1/Cod1 ATPase activity was blocked at 500 µM vanadate [20].

We further showed that *cta4^+^* loss leads to ER stress response and subsequent stimulation of intracellular calcium accumulation. It should be noted that calcium accumulation was recovered to wild-type levels in *cta4Δ* cells expressing *cta4* ^+^ (data not shown). Elevated Ca^2+^ levels promote activation of calcineurin, essential for *cta4Δ* survival. In animal cells, the inhibitors of SERCA pumps activate the entry of Ca^2+^ into the cytoplasm through plasma membrane channels, a mechanism known as capacitative calcium entry or CCE [9]. Inhibition of ER Ca^2+^-ATPases also leads to increase of mRNA levels for the ER stress marker proteins BiP/GRP78 [37]. Elevation of intracellular calcium was demonstrated in budding yeast, in response to deletion of Golgi Ca^2+^-ATPase Pmr1p [10] and ER stress [14]. In budding yeast, ER stress triggers Ca^2+^ influx through a plasma membrane HACS channel resulting in activation of calcineurin [14]. Indeed, the activity of HACS channel as judged from detection of Mg^2+^ insensitive component of ^45^Ca^2+^ uptake was significantly increased in *cta4Δ* (unpublished data).

It has been suggested recently that the activation of HACS is a major response to defects in the secretory, endosomal and vacuolar protein trafficking pathways [38]. Genome-wide approach in *S. cerevisiae* allowed the identification of group A mutants (HACS regulators) with spontaneous HACS activation and defects in endomembrane trafficking system, and group B mutants which require external stimuli (tunicamycin or α-factor) to activate Ca^2+^ influx. Importantly, our results place *cta4Δ* in group A, together with *pmr1* mutant. Notably, *spf1* and *pmc1* mutants belong to group B. It should be emphasized that loss of Spf1/Cod1 ATPase in *S. cerevisiae* did not result in changes in cellular calcium levels [20], activation of Ca^2+^ influx and HACS [38]. Thus, although Cta4p and Spf1/Cod1p share amino acid identity, both reside to ER and corresponding mutants display sensitivity towards extracellular calcium, they apparently have distinct functions in endomembrane trafficking system and calcium homeostasis. This study also provides direct biochemical evidence that specific calcineurin phosphatase activity is highly induced upon *cta4^+^* disruption. In this respect, it is noteworthy that mutant lacking Cta4 ATPase was sensitive to inhibition of calcineurin [18] indicating that activated calcineurin is essential for the survival of *cta4Δ* cells undergoing ER stress. This finding is in agreement with the demonstration of requirement of calcineurin for a survival of mutants with activated HACS [38]. Future study should identify the targets of calcineurin responsible for cell survival under ER stress in *S. pombe*. However, one of these targets has been already identified. We showed that *cta4*Δ mutant displays an increase in Ca^2+^-ATPase activity in vacuole membranes as compared to wild-type. Thus, induced calcium sequestration to vacuoles possibly compensates for the loss of the Cta4p from the ER, and contributes to lowering the cytoplasmic calcium. Also, we found that *cta4*Δ exhibits a decrease in activity of Ca^2+^/H^+^ antiporters. Our findings support the idea that elevated intracellular calcium in *cta4Δ* leads to activation of the calcineurin which, in turn, differentially regulates other Ca^2+^ transporters. This is consistent with results obtained for the *S. cerevisiae* Vcx1, a vacuolar H^+^/Ca^2+^ exchanger, which activity is inhibited by calcineurin [21], whereas the expression and activity of vacuolar calcium pump Pmc1p is stimulated by Ca^2+^ in calcineurin-dependent manner [22], [39]. The *S. cerevisiae* Golgi-localized Ca^2+^-ATPase Pmr1p was shown to be dependent on calcineurin [22] but to much less extent [6], [40]. In *S. pombe*, Pmr1p/Csp5p is localized to ER [33] and is likely responsible for remaining calcium transport in ER of *cta4Δ*. Our data suggest that vacuolar Ca^2+^-ATPase Pmc1p assumes a leading role in calcium homeostasis in *cta4Δ* genetic background. This suggests that vacuolar pump could serve as a backup system when Cta4 ATPase is missing and under calcium overload. Indeed, the *S. pombe pmc1* ^+^ was required for growth in medium in the presence of high extracellular CaCl_2_ [41]. It is likely that fission yeast vacuolar Pmc1p expression is activated by calcineurin in *cta4Δ*. This supposition is consistent with the demonstration of the induction of *PMC1* transcription in *S. cerevisiae* mutants lacking Spf1p/Cod1p P_5A_ATPase using ß-galactosidase reporter plasmid [20].

The data also established a critical role of Cta4p in maintaining the ER folding environment. The findings reported here indicate that *cta4Δ* mutant is under ER stress as revealed by the increased expression of the ER stress indicator, the chaperone BiP. The distribution of BiP in Golgi and vacuole membrane fractions isolated from *cta4Δ* mutant deserves special mention. Disruption of *cta4^+^* may affect not only BiP distribution but also that of other proteins of the secretory pathway. In agreement, it was demonstrated that loss of another P_5A_ ATPase, Spf1/Cod1p from *S. cerevisiae*, resulted in altered distribution of another ER membrane protein, Sec12 [42]. Additionally, a mutation in the P_5A_ ATPase gene MIA from Arabidopsis resulted in major changes in expression of genes involved in protein secretion [30]. Thus, one of physiological roles of P_5A_ ATPases is to control the proper functioning of secretory pathway and protein targeting. This finding is not without precedence, since deletion of *S. cerevisiae* Pmr1 Ca^2+^-ATPase also resulted in ER stress [43] and significant redistribution of enzyme activities and total protein in compartments of the secretory pathway [25].

Loss of *cta4^+^* resulted in ER stress and inability to cope with accumulation of unfolded proteins. Although the ER-associated protein degradation constitutes a main mechanism for elimination of unfolded protein, there is growing evidence for a vacuole participation in this process. A recent study in tobacco has shown that BiP is transported to the vacuole and that the ER export of BiP occurs via COPII-dependent transport to the Golgi apparatus [44]. It is likely that in *cta4Δ* vacuolar disposal of proteins could act in addition to ER-associated protein degradation to improve the efficiency of quality control throughout in the secretory pathway. The stimulation of vacuole Ca^2+^ transport observed in *cta4Δ* might be required to maximize vacuole functioning, besides of lowering cytoplasmic calcium levels. Thus, the vacuole shares a role in calcium homeostasis and quality control with ER.

Our results adds biochemical substantiation to the argument that Cta4p might transport Ca^2+^ or, at least, regulates Ca^2+^ transport in ER by influencing functional localization of Pmr1p in the ER. Although both alternatives remain to be investigated, the last one should not be the case for Spf1/Cod1p from *S. cerevisiae* since Pmr1p is localized to Golgi in this yeast [25].

In conclusion, we provide functional evidence of Ca^2+^ transport mediated by Ca^2+^-ATPase in fission yeast membranes. Our findings depict a crucial role of P_5A_-type Cta4 ATPase in Ca^2+^ transport in ER and regulation of Ca^2+^ influx system. Finally, our data provide a platform for future studies of the signaling network which encompasses the calcineurin and Ca^2+^-ATPases activity and controls the calcium homeostasis and the function of the endomembrane system in *S. pombe*.
